# Supplementary material for: ETV4 and ETV5 Orchestrate FGF-Mediated Lineage Specification and Epiblast Maturation during Early Mouse Development
Source: bioRxiv. 2024 Jul 24:2024.07.24.604964. Preprint. [Version 1] doi: 10.1101/2024.07.24.604964 (PMC11291132; doi:10.1101/2024.07.24.604964)

Simon et al Supplemental Figure 1

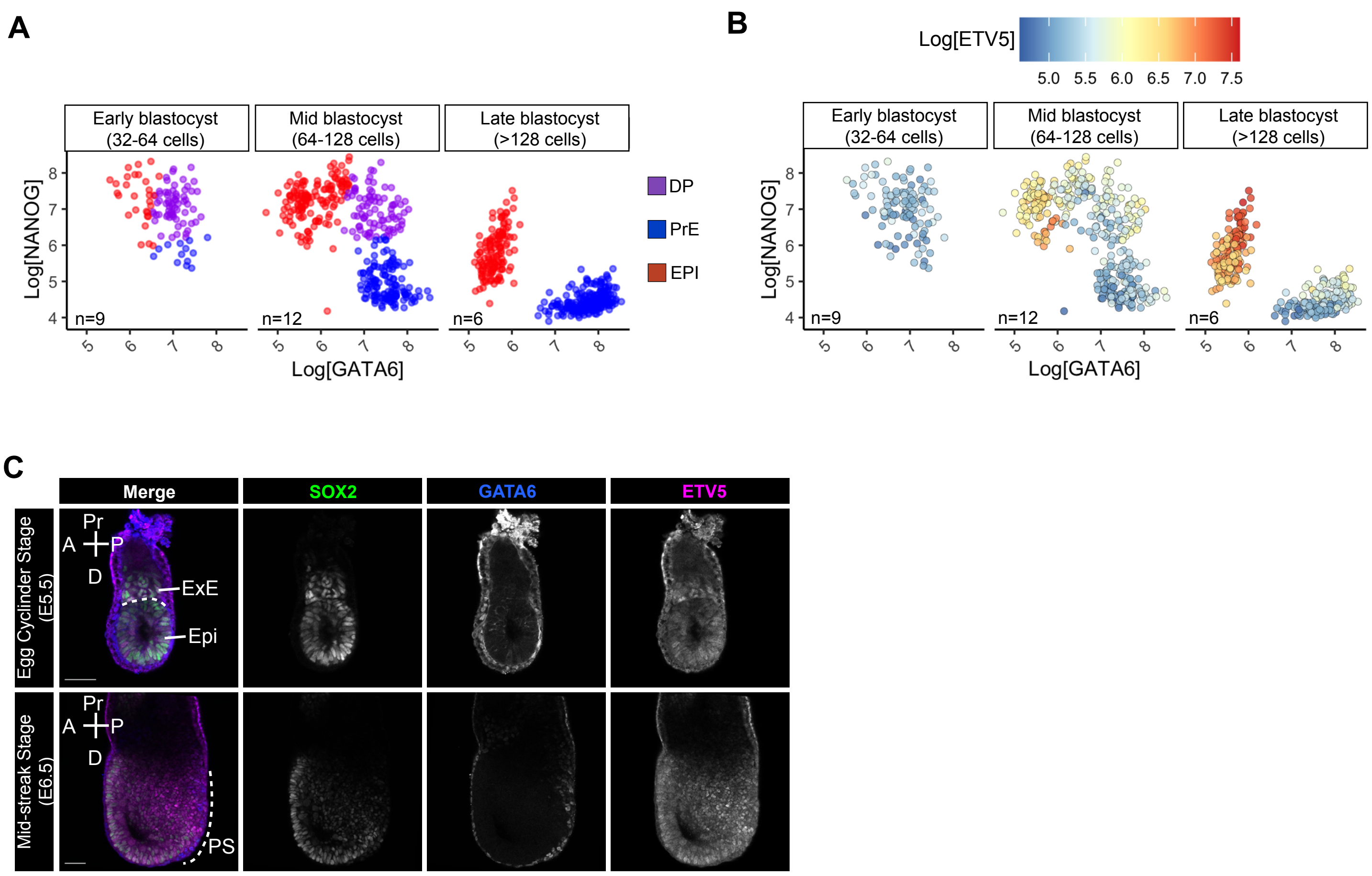

Simon et al Supplemental Figure 2

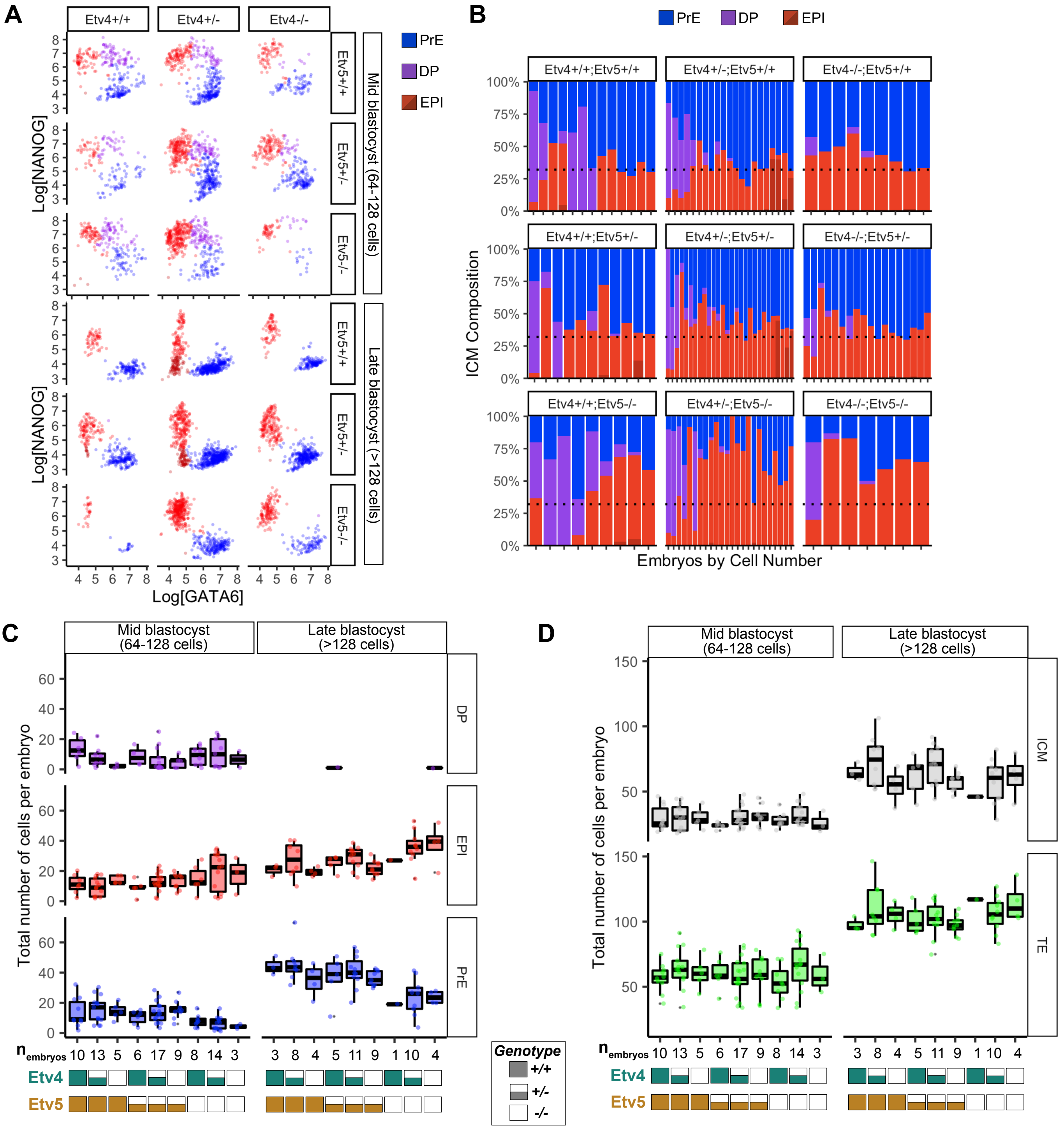

Simon et al Supplemental Figure 3

A

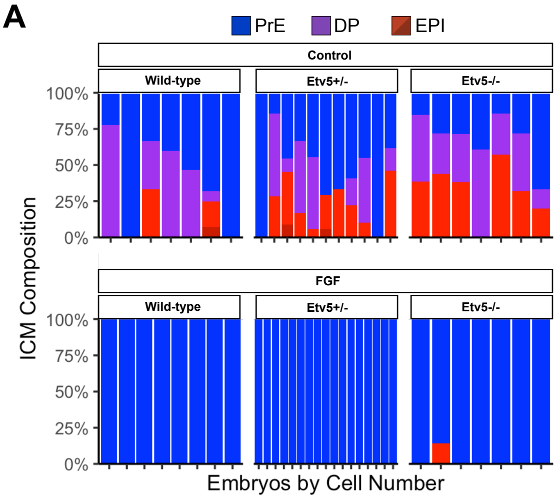

Simon et al Supplemental Figure 4

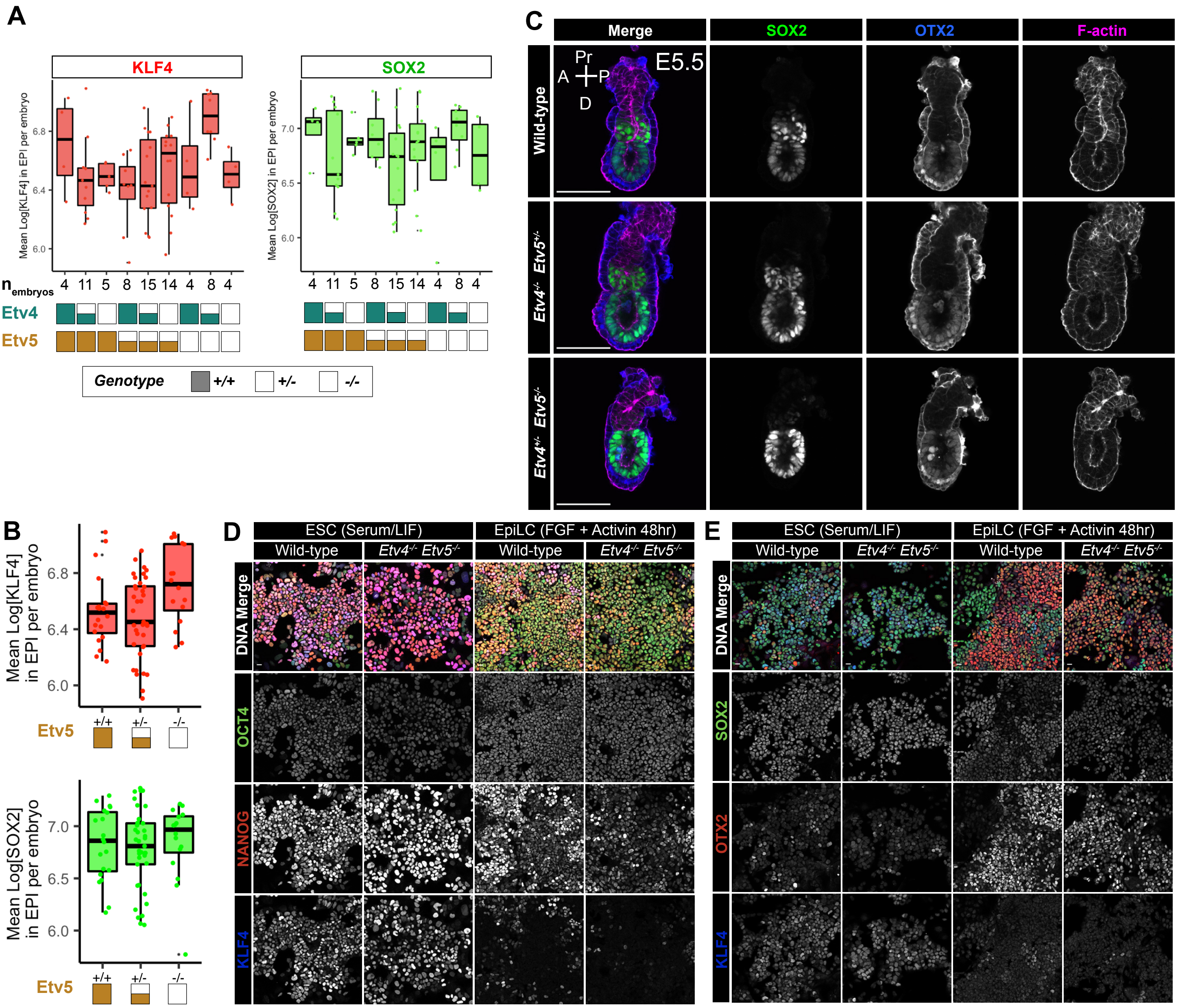

Simon et al Supplemental Figure 5

A

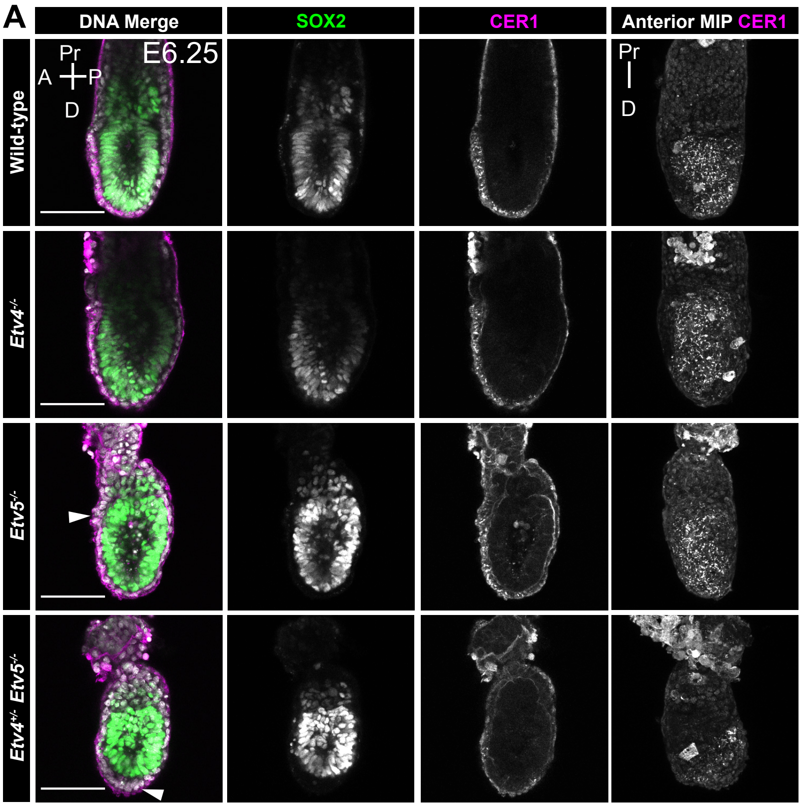

C

Observed *(Expected)* numbers: *Etv5*+/- intercross

Stage	Genotype *Etv5* +/+	+/-	-/-
**P21**	19 *(16)*	43 *(32)*	1 *(16)*

D

Observed *(Expected)* numbers: *Etv4*+/-;*Etv5*+/- intercross

Genotype									
Etv4	+/+	+/-	-/-	+/+	+/-	-/-	+/+	+/-	-/-
Etv5	+/+	+/+	+/+	+/-	+/-	+/-	-/-	-/-	-/-
**P21**	5 *(2)*	4 *(2)*	1 *(2)*	4 *(4)*	14 *(7)*	0 *(4)*	0 *(2)*	0 *(4)*	0 *(2)*

B

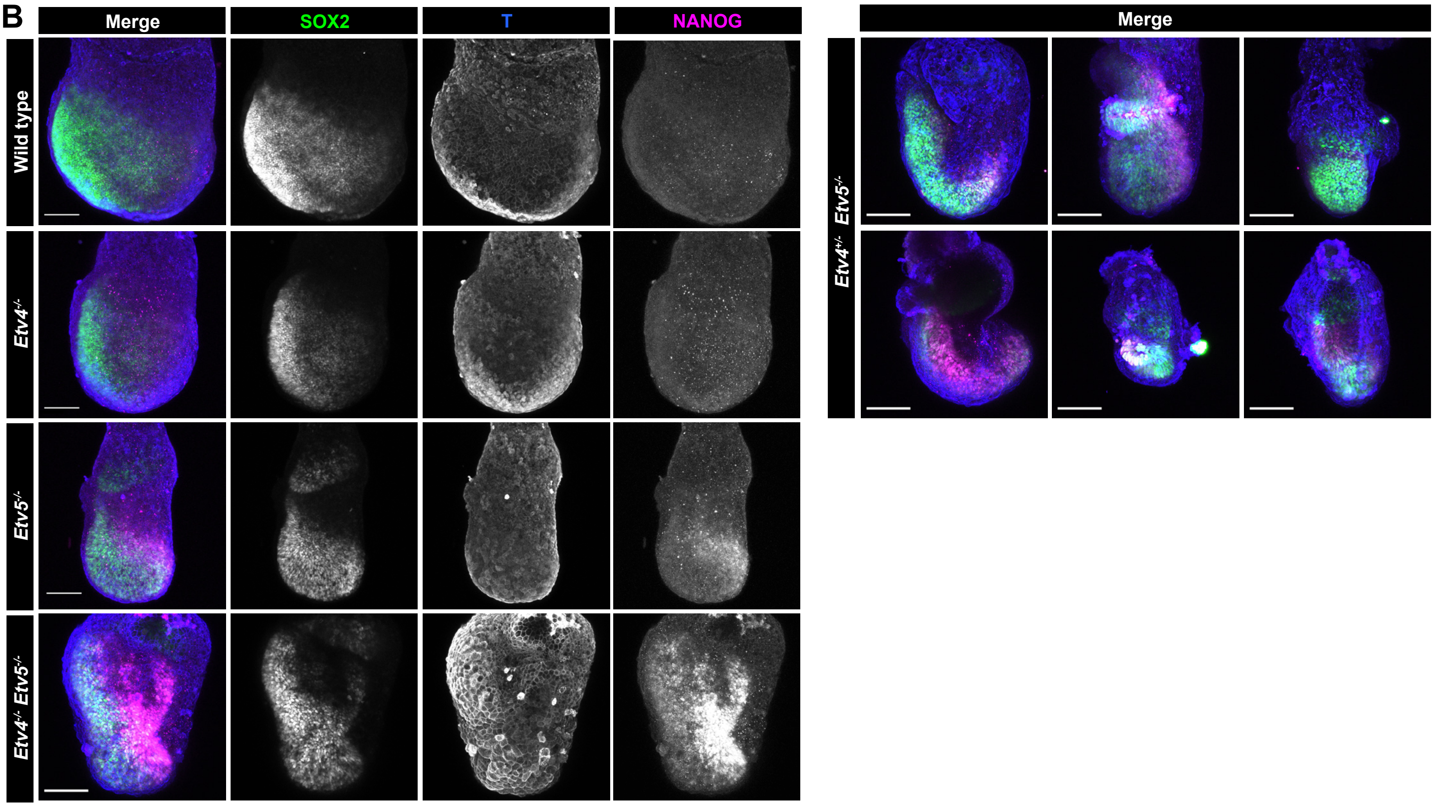

Supplement: Supplement 1 — Supplemental figure 1: Etv4 and Etv5 expression during mouse embryonic development (related to Figure 1) (A) Quantification of NANOG and GATA6 levels in blastocyst (from Figure 1B). Clustering into double positive (DP; NANOG+;GATA6+), epiblast (EPI; NANOG+;GATA6−) and primitive endoderm (PrE; NANOG−;GATA6+) (B) Heatmap of ETV5 levels in blastocyst compared to NANOG and GATA6 levels (from Figure 1B) (C) Confocal images of immunofluorescence immunostaining of SOX2, GATA6 and ETV5 in postimplantation stage stage mouse embryos. Scale bars 50μm. Anterior (A); posterior (P); proximal (Pr); distal (D); extraembryonic-ectoderm (ExE); epiblast (Epi); PS (Primitive streak). Supplemental figure 2: Loss of Etv5 compromises the formation of PrE (related to figure 2) (A) Quantification of NANOG and GATA6 levels in an Etv4;Etv5 allelic series of mutant embryos (from Figure 2A,B). Clustering into double positive (DP; NANOG+;GATA6+), primitive endoderm (PrE; NANOG−;GATA6+), epiblast (EPI; NANOG+;GATA6− light red, NANOG−;GATA6− dark red). (B) Quantification of inner cell mass (ICM) lineage composition in an allelic series of Etv4;Etv5 mutant embryos. Individual embryos shown and ordered by ascending cell number. Dotted line repressends mean wild-type EPI:PrE composition by late blastocyst stage. (C) Total number of cells per embryo in EPI and PrE lineages (D) Total number of cells per embryo in ICM and TE lineages. Supplemental figure 3: Mechanism of Etv5 action on ICM cell fate decision (related to figure 3) (A) Quantification of inner cell mass (ICM) lineage composition in control and FGF treated wild-type, Etv5+/− and Etv5−/− embryos (related to Figure 3B). Double positive (DP; NANOG+GATA6+), primitive endoderm (PrE; NANOG−;GATA6+), epiblast (EPI; NANOG+;GATA6− light red, NANOG−;GATA6− dark red). Individual embryos shown and ordered by ascending cell number. Supplemental figure 4: Loss of Etv4/5 causes a delay in the progression of pluripotency (A) Quantification of KLF4 and SOX2 [file media-1.pdf]
